# Perioperative Safety Considerations and Surgical Outcomes Associated With GLP-1-Based Therapies: A Systematic Review

**DOI:** 10.7759/cureus.116901

**Published:** 2026-09-25

**Authors:** Mayte Rodrigues da Cunha, Marco Túlio Rodrigues da Cunha

**Affiliations:** 1 Department of Research, Saint James School of Medicine, Kingstown, VCT; 2 Department of Plastic Surgery, Universidade Federal do Triângulo Mineiro, Uberaba, BRA

**Keywords:** anesthesia, aspiration risk, gastric emptying, glp-1 receptor agonists, perioperative care, preoperative fasting, residual gastric contents, semaglutide

## Abstract

Glucagon-like peptide-1 receptor agonists (GLP-1 RAs) are increasingly prescribed for the management of type 2 diabetes mellitus and obesity. Their effects on gastric emptying have raised concerns in the perioperative setting, where standard fasting protocols are used to reduce aspiration risk. However, the clinical significance of altered gastric physiology and its implications for perioperative management remain uncertain.

A systematic review was conducted in accordance with the Preferred Reporting Items for Systematic Reviews and Meta-Analyses (PRISMA) guidelines. The protocol was prospectively registered in the International Prospective Register of Systematic Reviews (PROSPERO) (registration number: CRD420261289189). A comprehensive literature search identified studies evaluating perioperative gastric physiology and procedural safety outcomes associated with GLP-1 RA use in adults undergoing procedures requiring sedation or general anesthesia. Outcomes included delayed gastric emptying, residual gastric contents, aspiration or regurgitation events, and perioperative management adaptations. Sixteen studies met the inclusion criteria, comprising seven observational studies and nine case reports or case series. Observational evidence demonstrated an association between GLP-1 RA use, particularly semaglutide, and increased residual gastric contents or delayed gastric emptying despite adherence to standard fasting protocols. These findings were identified primarily through gastric ultrasound and endoscopic assessment. In contrast, clinically apparent aspiration or regurgitation events were uncommon and were reported predominantly in case-based literature. Larger observational studies did not demonstrate a clear increase in aspiration or broader perioperative complications. Identification of retained gastric contents also influenced clinical management in several reports, resulting in changes to anesthetic planning, additional gastric assessment, or postponement of elective procedures.

GLP-1 RA therapy is associated with alterations in gastric physiology that may challenge the assumptions underlying standard preoperative fasting protocols. Although increased residual gastric contents are consistently reported, current evidence does not establish a corresponding increase in clinically significant aspiration events. Individualized preoperative risk assessment, including consideration of medication exposure and selective use of gastric ultrasound, may assist perioperative decision-making. Further prospective studies are needed to quantify aspiration risk and establish evidence-based perioperative management strategies.

## Introduction and background

Glucagon-like peptide-1 receptor agonists (GLP-1 RAs) have rapidly transformed the management of type 2 diabetes mellitus and obesity. Originally developed for glycemic control, these agents have demonstrated substantial benefits in weight reduction and cardiometabolic risk modification, leading to a marked expansion in their use across diverse patient populations. As a result, an increasing number of surgical patients now present for elective and urgent procedures while actively receiving GLP-1 RA therapy [1-6].

GLP-1 RAs exert their metabolic effects through multiple mechanisms, including enhancement of glucose-dependent insulin secretion, suppression of glucagon release, central appetite regulation, and modulation of gastrointestinal motility. While these mechanisms contribute to improved postprandial glycemic control and early satiety, they raise potential concerns in the perioperative setting, where impaired gastric emptying may increase residual gastric contents despite adherence to standard fasting guidelines [1-6].

Pulmonary aspiration of gastric contents during anesthesia is a rare but potentially catastrophic complication associated with significant morbidity and mortality. Current perioperative fasting protocols are designed to minimize this risk; however, these guidelines presume normal gastric physiology. Conditions associated with gastroparesis or delayed gastric emptying may compromise the reliability of standard fasting intervals, placing patients at increased risk even after appropriate preoperative preparation. In this context, the growing use of GLP-1 RAs has prompted renewed attention to their potential impact on perioperative safety [7-10].

Over the past several years, emerging evidence, including case reports, observational studies, gastric ultrasound investigations, and endoscopic assessments, has suggested an association between GLP-1 RA use and increased residual gastric contents in fasting patients [11-16]. These findings have generated significant clinical uncertainty and variability in perioperative management, with some institutions recommending prolonged medication discontinuation prior to surgery, while others advocate for individualized risk assessment or continuation of therapy [11-16]. The resulting uncertainty reflects the limited and heterogeneous nature of the available evidence [1-16].

Despite growing concern, the available literature remains fragmented, encompassing diverse study designs, patient populations, surgical contexts, and outcome measures [1-16]. The true magnitude of perioperative risk, the clinical significance of delayed gastric emptying, and the optimal duration of medication interruption, if any, remain unclear [1-16]. A comprehensive synthesis of existing evidence is therefore necessary to inform perioperative decision-making and guide future research.

Accordingly, this systematic review aims to evaluate perioperative safety considerations and surgical outcomes associated with GLP-1 RA use, with particular focus on residual gastric contents, aspiration-related events, and perioperative management implications.

## Review

Methods

Protocol and Registration

This systematic review was conducted in accordance with the Preferred Reporting Items for Systematic Reviews and Meta-Analyses (PRISMA) 2020 guidelines [17]. The protocol was prospectively registered in the International Prospective Register of Systematic Reviews (PROSPERO) under registration number CRD420261289189.

Search Strategy

A comprehensive literature search was conducted using PubMed/Medical Literature Analysis and Retrieval System Online (MEDLINE) to identify studies evaluating perioperative safety considerations and surgical outcomes associated with GLP-1 RA use. The search strategy combined Medical Subject Headings (MeSH) and free-text terms related to GLP-1 RAs, perioperative care, gastric emptying, aspiration, and surgical outcomes. Studies published from database inception through January 2026 were considered. Only articles published in English were included. Reference lists of eligible studies were manually screened to identify additional relevant publications.

Eligibility Criteria

Studies were selected according to predefined inclusion and exclusion criteria based on the Population, Exposure, Comparator, and Outcomes (PECO) framework [18].

Population: Adults (>18 years) undergoing elective or urgent surgical or procedural interventions requiring sedation or general anesthesia were eligible. Studies including patients with type 2 diabetes mellitus or obesity receiving GLP-1 RA therapy were included.

Exposure: The exposure of interest was treatment with a GLP-1 RA, including but not limited to semaglutide, liraglutide, dulaglutide, exenatide, and lixisenatide.

Comparators: Comparator groups, when applicable, included individuals not exposed to GLP-1 RAs, placebo, standard care, or pre-post comparisons within the same participants.

Outcomes: Primary outcomes included evidence of delayed gastric emptying, increased residual gastric contents, aspiration or regurgitation events, and perioperative complications related to gastric physiology. Secondary outcomes included gastric ultrasound findings, symptom burden, perioperative management strategies, and surgical delay or cancellation.

Study designs: Randomized controlled trials, non-randomized observational studies, prospective and retrospective cohort studies, case series, and case reports were eligible for inclusion.

Exclusion criteria: Studies involving pediatric populations (<18 years), animal models, non-perioperative settings, editorials, narrative reviews, conference abstracts without full-text availability, and non-original research were excluded.

Study Selection

All records identified through the literature search were imported into Rayyan (Qatar Computing Research Institute, Doha, Qatar) for study management. Following removal of duplicates, two reviewers independently screened titles and abstracts for eligibility, followed by full-text review of potentially relevant studies. Discrepancies were resolved through discussion and consensus between the reviewers. The study selection process is illustrated in the PRISMA flow diagram (Figure 1). A total of 333 records were identified through the database search. After title and abstract screening, 317 records were excluded for not meeting the predefined eligibility criteria. Sixteen articles met the eligibility criteria and were included in the final qualitative synthesis. No duplicate records were identified. The study selection process is summarized in the PRISMA flow diagram (Figure 1).

**Figure 1:**
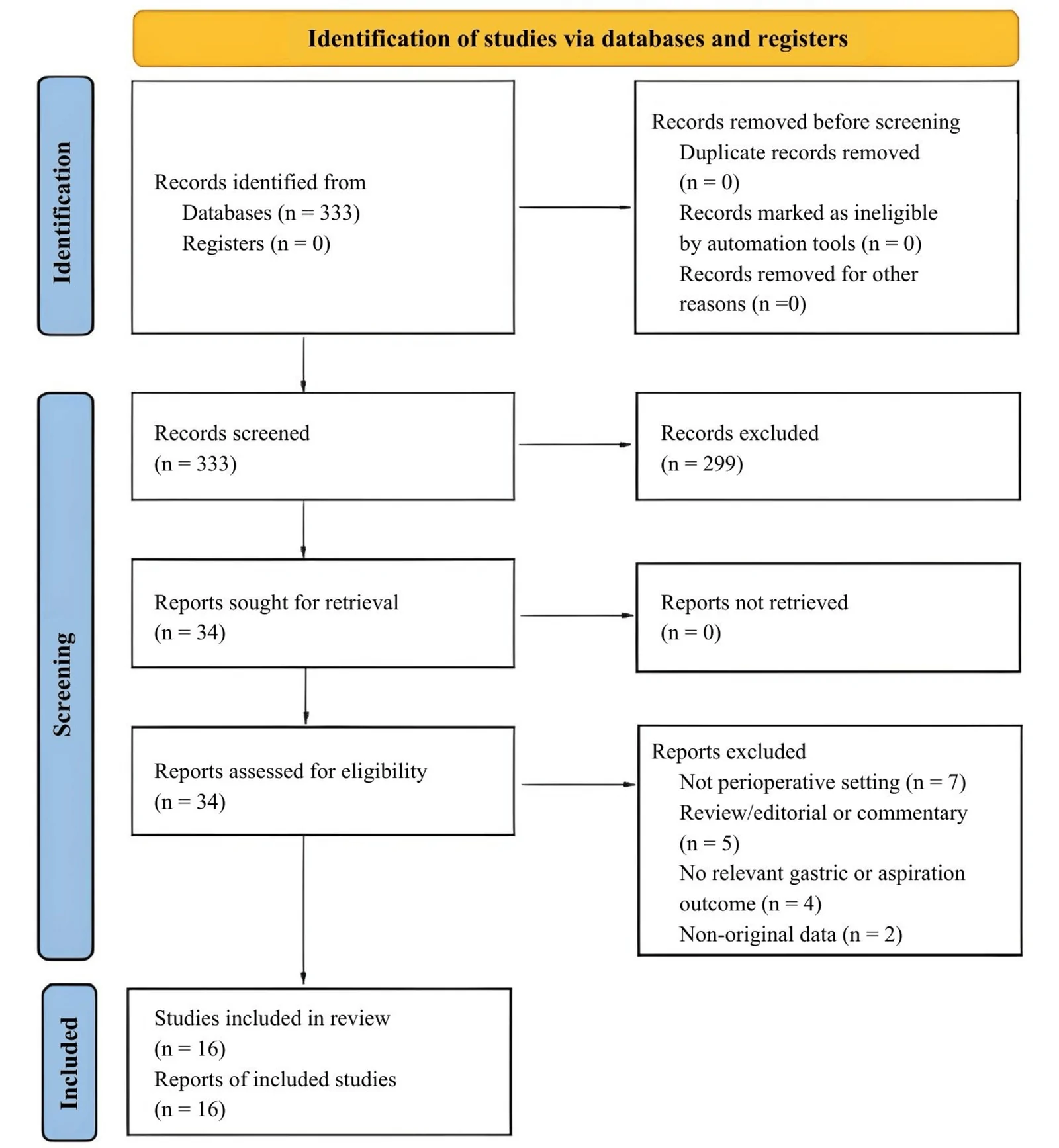
PRISMA 2020 flow diagram of study selection PRISMA: Preferred Reporting Items for Systematic Reviews and Meta-Analyses

Data Extraction

Data were extracted using a standardized data collection form. Extracted variables included study design, publication year, country, patient characteristics, type of GLP-1 RA, duration and timing of exposure, perioperative context, assessed outcomes, and principal findings related to gastric emptying and perioperative safety.

Risk of Bias Assessment

Given the heterogeneity of the included study designs, risk of bias was assessed qualitatively according to study type. Observational studies were evaluated with attention to study design, participant selection, exposure and outcome assessment, and potential confounding. Case reports and case series were assessed with attention to reporting completeness, clinical detail, and potential confounding. Given the heterogeneity of the evidence base, no formal quantitative risk-of-bias score was calculated.

Data Synthesis

Owing to substantial heterogeneity in study design, outcome measures, and perioperative contexts, a meta-analysis was not performed. Findings were synthesized qualitatively using a narrative approach, with results grouped according to outcome categories and perioperative relevance.

Results

Study Characteristics

The 16 included studies consisted of seven observational studies and nine case reports or case series [1-16]. The observational evidence included prospective and retrospective studies evaluating gastric contents, perioperative outcomes, and the relationship between GLP-1 RA exposure and gastric emptying [2,3,5,7,11,14,16]. The remaining literature consisted of individual case reports and small case series describing retained gastric contents, delayed gastric emptying, regurgitation, aspiration, or changes in perioperative management [1,4,6,8-10,12,13,15].

Study populations included adults receiving GLP-1 RA therapy for type 2 diabetes mellitus or weight management [1-16]. Semaglutide was the most frequently evaluated individual agent, although several studies examined GLP-1 RAs as a therapeutic class [1-16]. The procedural settings were heterogeneous and included elective surgery under general anesthesia, upper gastrointestinal endoscopy, hip and knee arthroplasty, electroconvulsive therapy, preoperative gastric ultrasound assessment, and fasting assessments in volunteers [1-16].

The outcomes evaluated across the included literature also varied considerably [1-16]. Observational studies primarily assessed residual gastric volume, the presence of solid gastric contents, gastric ultrasound findings, or perioperative complications [2,3,5,7,11,14,16]. Case-based evidence provided additional descriptions of clinically apparent regurgitation or aspiration, retained gastric contents despite fasting, and alterations in anesthetic or procedural management [1,4,6,8-10,12,13,15]. The characteristics and principal findings of all included studies are summarized in Table 1.

**Table 1:** Characteristics of included studies T2DM: type 2 diabetes mellitus; GLP-1 RA: glucagon-like peptide-1 receptor agonist

Author (Year)	Study Design	Population	GLP-1 Receptor Agonist	Perioperative / Procedural Context	Main Findings
Klonoff et al. (2024) [14]	Large observational cohort	Adults with T2DM undergoing surgery	GLP-1 RAs (class)	General anesthesia	No increased peri- or postoperative complications or aspiration events
Nersessian et al. (2024) [11]	Prospective observational	Adults receiving semaglutide	Semaglutide	Elective surgery	Increased residual gastric content on preoperative ultrasound
Santos et al. (2024) [2]	Retrospective observational	Adults undergoing endoscopy	Semaglutide	EGD under sedation/anesthesia	Increased residual gastric content associated with recent semaglutide use
Mahmoud et al. (2025) [7]	Multicenter observational	Patients with T2DM	GLP-1 RAs (class)	Hip and knee arthroplasty	No perioperative aspiration events observed
Sherwin et al. (2023) [5]	Prospective observational	Volunteers without obesity	Semaglutide	Simulated perioperative fasting	Higher prevalence of solid gastric contents
Wolla et al. (2025) [16]	Prospective cohort	Fasted diabetic surgical patients	GLP-1 RAs (class)	Elective surgery	Increased gastric volume and full stomach risk
Queiroz et al. (2023) [13]	Case report	Adult patient	Semaglutide	General anesthesia	Intraoperative regurgitation identified on CT
Espinoza & Antongiorgi (2024) [4]	Case report	Adult patient undergoing ECT	Semaglutide	Electroconvulsive therapy	Management concerns due to delayed gastric emptying
Klein & Hobai (2023) [15]	Case report	Adult patient	Semaglutide	Upper endoscopy	Aspiration of gastric contents despite prolonged fasting
Raven et al. (2023) [1]	Case report	Adult patient	GLP-1 RAs (class)	Surgery under general anesthesia	Delayed gastric emptying with perioperative use
Gulak & Murphy (2023) [10]	Case report	Adult patient	Semaglutide	General anesthesia	Regurgitation despite appropriate fasting
Girón-Arango & Perlas (2024) [8]	Case report	Adult patient with T2DM	GLP-1 RAs (class)	Preoperative ultrasound	Ultrasound-guided management change
Kittner et al. (2023) [12]	Case series (3 cases)	Adults	GLP-1 RAs (class)	Elective surgery	Retained gastric contents after adequate fasting
Wilson et al. (2023) [6]	Case series (2 cases)	Adults	GLP-1 RAs (class)	Elective surgery	Particulate gastric contents identified
Queiroz et al. (2025) [3]	Observational	Adult volunteers	Semaglutide	Fasting assessment	Higher prevalence of full stomach on ultrasound
Hodgson et al. (2024) [9]	Case report	Adult patient	Semaglutide	Preoperative assessment	Ultrasound altered anesthetic management

Primary Outcomes

The primary outcomes of interest were delayed gastric emptying, increased residual gastric contents, and perioperative aspiration or regurgitation events. Across the observational literature, multiple studies demonstrated an association between GLP-1 RA exposure and increased residual gastric contents despite adherence to preprocedural fasting requirements [2,3,5,11,16]. These findings were identified using objective methods, including point-of-care gastric ultrasound and endoscopic assessment [2,3,5,11,16].

Semaglutide was particularly prominent in the evidence evaluating residual gastric contents. Nersessian et al. reported increased residual gastric content among patients receiving perioperative semaglutide using preoperative gastric ultrasound [11]. Santos et al. similarly identified an association between recent semaglutide exposure and increased residual gastric content during endoscopic assessment [2]. Sherwin et al. demonstrated a higher prevalence of residual gastric solids on ultrasound among volunteers recently started on semaglutide [5], while Queiroz et al. reported a higher prevalence of a full stomach during fasting assessment among semaglutide users [3]. Wolla et al. further demonstrated increased preoperative gastric volume and full stomach risk among fasted diabetic surgical patients receiving GLP-1 RAs [16].

The timing of medication interruption also emerged as a clinically relevant consideration [2,11]. Santos et al. and Nersessian et al. evaluated the relationship between semaglutide interruption and residual gastric contents, with shorter interruption intervals associated with a greater likelihood of retained gastric contents [2,11]. However, differences in study populations, exposure definitions, interruption intervals, and methods used to assess gastric contents limited direct comparison between studies [2,11].

Clinically apparent aspiration and regurgitation were substantially less frequently reported than increased residual gastric contents. Gulak and Murphy described regurgitation during anesthesia in an appropriately fasted patient receiving semaglutide [10]. Queiroz et al. reported perioperative regurgitation in a fasting patient using semaglutide, with retained gastric contents demonstrated by imaging [13]. Klein and Hobai reported pulmonary aspiration associated with semaglutide despite prolonged fasting [15]. Additional case-based literature documented retained or particulate gastric contents after appropriate fasting without necessarily demonstrating a clinically significant aspiration event [6,12]. In contrast, larger observational studies did not demonstrate a clear increase in aspiration or broader perioperative complications associated with GLP-1 RA use [7,14].

Taken together, the included evidence demonstrated a more consistent association between GLP-1 RA exposure and altered gastric contents than between exposure and clinically significant aspiration events [1-16]. A summary of the principal perioperative gastric findings and their clinical implications is presented in Table 2.

**Table 2:** Summary of findings GLP-1 RA: glucagon-like peptide-1 receptor agonist

Perioperative Outcome	Summary of Findings	Evidence Source
Delayed gastric emptying	GLP-1 RA use was associated with delayed gastric emptying despite adherence to standard fasting guidelines.	Observational studies [2,3,5,11,16]
Increased residual gastric contents	Higher prevalence of solid or increased-volume gastric contents identified on gastric ultrasound or endoscopic assessment, particularly with recent semaglutide dosing.	Observational studies [2,3,5,11,16]
Relationship to dosing intervals	Shorter interruption intervals were more frequently associated with increased residual gastric contents compared with longer discontinuation periods.	Observational studies [2,11]
Aspiration or regurgitation events	Perioperative aspiration or regurgitation events were rare and primarily reported in case reports involving appropriately fasted patients.	Case reports and case series [10,13,15]
Symptom-gastric content correlation	Gastrointestinal symptoms (e.g., nausea, early satiety) did not reliably predict objective gastric findings.	Observational studies [2,3,11]
Point-of-care gastric ultrasound	Gastric ultrasound frequently identified full stomachs and aided perioperative risk stratification.	Observational studies and case reports [3,5,8,9,11,16]
Perioperative management impact	Identification of increased gastric contents led to modification of anesthetic technique, procedure delay, or case postponement in several reports.	Observational studies and case reports [2,4,8,9,12]

Secondary Outcomes

Secondary outcomes included gastric ultrasound findings, gastrointestinal symptoms, perioperative management strategies, and procedural delay or modification. Point-of-care gastric ultrasound was used in several studies to assess gastric contents in patients receiving GLP-1 RAs and frequently identified solid contents, increased gastric volume, or findings consistent with a full stomach [3,5,8,9,11,16]. These findings demonstrate the potential utility of gastric ultrasound as an adjunct to standard preoperative assessment when uncertainty regarding gastric contents remains [3,5,8,9,11,16].

Gastric ultrasound findings also influenced clinical management in the case-based literature. Girón-Arango and Perlas described a change in perioperative management following preoperative ultrasound assessment in a patient receiving a GLP-1 RA [8]. Hodgson et al. similarly reported that point-of-care ultrasound altered anesthetic management in a patient taking semaglutide before surgery [9]. Across the broader literature, identification or concern for retained gastric contents resulted in changes to anesthetic planning, additional gastric assessment, or postponement of procedures in selected patients [4,8,9,12].

Gastrointestinal symptoms were not consistently predictive of objective gastric contents. Studies evaluating symptoms alongside gastric assessment demonstrated that the absence of symptoms did not reliably exclude increased residual gastric contents [2,3,11]. This finding is clinically relevant because patients may demonstrate altered gastric physiology despite appearing asymptomatic and adhering to conventional fasting instructions [2,3,11].

Perioperative management strategies varied considerably across the included studies and reflected the heterogeneity of the available evidence. Reported approaches included temporary interruption of GLP-1 RA therapy, additional assessment of gastric contents, modification of anesthetic planning, and postponement of elective procedures when a full stomach was identified or suspected [2,4,8,9,12]. This variability illustrates the practical impact of uncertainty surrounding GLP-1 RA use in the perioperative setting and the absence of a single management strategy consistently applied across the included literature [1-16].

Discussion

Principal Findings

This systematic review synthesizes the available evidence regarding the perioperative effects of GLP-1 RA therapy on gastric physiology and procedural safety. Across the 16 included studies, the most consistent finding was an association between GLP-1 RA exposure and delayed gastric emptying or increased residual gastric contents despite adherence to standard fasting recommendations [1-6,8,9,11-13,15,16]. These findings were demonstrated using objective assessments, including gastric ultrasound, endoscopic evaluation, and imaging [2,3,5,8,9,11,13,16].

The strongest and most reproducible signal in the literature concerned retained gastric contents rather than clinically significant aspiration. Several observational studies identified increased gastric volume, solid gastric contents, or a higher prevalence of a full stomach among patients receiving semaglutide or other GLP-1 RAs [2,3,5,11,16]. In contrast, aspiration and regurgitation events were uncommon and were reported primarily through individual case reports [10,13,15]. Larger observational cohorts did not demonstrate a clear increase in perioperative aspiration or broader complication rates [7,14].

This distinction is important because the presence of increased residual gastric contents represents a physiologic risk marker, while pulmonary aspiration is a comparatively rare clinical event [2,3,5,7,10,11,13-16]. The available evidence therefore supports concern regarding altered gastric physiology in GLP-1 RA users but does not establish a proportional increase in clinically significant aspiration across all exposed patients [2,3,5,7,11,14,16].

The evidence also suggests that the timing of the most recent GLP-1 RA dose may influence gastric findings. Studies evaluating semaglutide interruption intervals reported greater residual gastric contents with shorter intervals between the last dose and the procedure [2,11]. However, differences in study design, patient populations, assessment methods, and interruption periods limit the ability to define a single optimal discontinuation interval [2,11].

Clinical Implications

These findings have important implications for perioperative assessment and anesthetic planning. In patients receiving GLP-1 RAs, several studies have demonstrated retained gastric contents despite compliance with conventional fasting instructions [2,3,5,6,11,12,16].

As a result, fasting duration alone may not reliably identify all patients with residual gastric contents [2,3,5,6,11,12,16]. This is particularly relevant because increased gastric contents have also been reported in patients who were asymptomatic at the time of assessment [2,3,11]. Gastrointestinal symptoms such as nausea, early satiety, or bloating may therefore contribute useful clinical information but should not be considered a definitive surrogate for gastric contents [2,3,11].

Point-of-care gastric ultrasound may provide additional information when the presence of gastric contents is uncertain. Multiple studies and case reports demonstrated its ability to identify solid contents or increased gastric volume and to influence perioperative management [3,5,8,9,11,16]. In selected patients, ultrasound findings contributed to modification of anesthetic technique, additional precautions, or postponement of elective procedures [8,9].

The evidence does not support treating every patient receiving a GLP-1 RA as having the same degree of perioperative risk [1-16]. Exposure characteristics, medication timing, indication for treatment, gastrointestinal symptoms, and the urgency and type of procedure may all influence clinical decision-making [1-16]. The heterogeneity of the available literature supports an individualized approach rather than a uniform assumption of increased aspiration risk in all patients receiving these medications [1-16].

Comparison With Existing Guidelines and Literature

The findings of this review are consistent with growing concern regarding the effect of GLP-1 RAs on perioperative gastric physiology [2,3,5,11,16]. The observational studies included in this review repeatedly demonstrated measurable differences in gastric contents among exposed patients, particularly among patients receiving semaglutide [2,3,5,11,16]. At the same time, the available evidence does not demonstrate a similarly consistent increase in clinically significant perioperative aspiration [7,14].

This discrepancy between physiologic findings and clinical outcomes has important implications for perioperative management. Increased residual gastric contents may plausibly increase aspiration risk, but the rarity of aspiration events makes this outcome difficult to evaluate using small observational studies and case-based literature [6,7,10,12-15]. Case reports are useful for identifying potential safety signals, but they cannot establish incidence or quantify risk within the wider population [6,10,12,13,15].

The current evidence therefore supports increased awareness and risk assessment without providing definitive evidence for a single universal management strategy. The variability in medication interruption periods, fasting protocols, patient characteristics, and gastric assessment methods across studies further limits direct comparison and contributes to uncertainty regarding the most appropriate perioperative approach [1-16].

These findings also help explain why perioperative recommendations have evolved as new evidence has emerged, with recent multidisciplinary guidance emphasizing individualized risk assessment rather than a uniform approach to perioperative GLP-1 RA management [19]. Early concern was driven largely by pharmacologic effects on gastric emptying and individual reports of retained gastric contents or aspiration, whereas more recent observational data have provided a broader view of physiologic changes and clinical outcomes [2,3,7,11,14,16]. Continued refinement of perioperative guidance will likely depend on higher quality prospective evidence capable of linking objective gastric findings with clinically meaningful outcomes.

Limitations

This review has several limitations. First, the literature search was conducted exclusively using PubMed/MEDLINE. Consequently, potentially relevant studies indexed only in other databases may not have been identified.

Second, the available evidence was heterogeneous with respect to study design, patient population, type of GLP-1 RA, indication for treatment, medication interruption interval, procedural setting, fasting protocol, and method used to assess gastric contents [1-16]. This heterogeneity prevented meaningful quantitative pooling and limits direct comparison across studies [1-16].

Third, a substantial proportion of the available evidence consisted of case reports and case series [1,4,6,8-10,12,13,15]. Because these reports describe individual patients or small numbers of patients without comparative groups, they could not be used to estimate incidence or establish causality within the wider population [1,4,6,8-10,12,13,15].

Fourth, several studies focused primarily on surrogate outcomes such as residual gastric volume or the presence of solid gastric contents rather than clinically significant outcomes such as pulmonary aspiration [2,3,5,11,16]. Although these physiologic findings may be relevant to aspiration risk, the relationship between increased residual gastric contents and the absolute incidence of aspiration remains uncertain [2,3,5,7,11,14,16].

Finally, the case-based literature may be subject to publication bias, potentially resulting in overrepresentation of clinically notable adverse events relative to uncomplicated exposures.

Future Directions

Future research should prioritize prospective, adequately powered studies evaluating standardized perioperative outcomes in patients receiving GLP-1 RAs. Particular attention should be given to the relationship between medication timing, dose, duration of therapy, gastrointestinal symptoms, and objectively measured gastric contents.

Studies using standardized gastric ultrasound protocols may help determine whether objective gastric assessment can reliably identify patients at increased perioperative risk and reduce unnecessary delays or cancellations. Larger multicenter cohorts will also be necessary to establish the true incidence of aspiration and determine whether increased residual gastric contents translate into clinically meaningful adverse outcomes.

Further research should also evaluate whether perioperative risk differs according to the specific GLP-1 RA, indication for treatment, dosing schedule, or stage of therapy. Such evidence would help determine whether medication interruption should be individualized rather than applied uniformly.

Ultimately, higher quality evidence is needed to support perioperative strategies that balance patient safety with the potential consequences of unnecessary medication discontinuation or procedural delay.

## Conclusions

GLP-1 RA therapy is associated with delayed gastric emptying and increased residual gastric contents in the perioperative setting, even among patients adhering to standard fasting guidelines. Although aspiration events appear uncommon, the potential for retained gastric contents has important implications for anesthetic planning and procedural safety.

Current evidence supports incorporating GLP-1 RA use into preoperative risk assessment and considering selective use of gastric ultrasound in higher-risk patients. Further prospective studies are needed to better define clinically meaningful aspiration risk and establish standardized perioperative management strategies.
